# Differential Repeated Sprinting Training in Youth Basketball Players: An Analysis of Effects According to Maturity Status

**DOI:** 10.3390/ijerph191912265

**Published:** 2022-09-27

**Authors:** Jorge Arede, John F. T. Fernandes, Wolfgang I. Schöllhorn, Nuno Leite

**Affiliations:** 1 Department of Sports Sciences, Exercise and Health, University of Trás-os-Montes and Alto Douro, 5001-801 Vila Real, Portugal; 2 School of Education, Polytechnic Institute of Viseu, 3504-501 Viseu, Portugal; 3 Department of Sports, Higher Institute of Educational Sciences of the Douro, 4560-708 Penafiel, Portugal; 4 School of Sports Sciences, Universidad Europea de Madrid, Campus de Villaviciosa de Odón, 28670 Villaviciosa de Odón, Spain; 5 School of Sport and Health Sciences, Cardiff Metropolitan University, Cardiff CF23 6XD, UK; 6 Institute of Sport Science, Training and Movement Science, University of Mainz, 55122 Mainz, Germany; 7 Research Center in Sports Sciences, Health Sciences and Human Development, CIDESD, University of Trás-os-Montes and Alto Douro, 5001-801 Vila Real, Portugal

**Keywords:** team sports, variation, movement variability, puberty, adolescence, growth, maturation, bilateral asymmetry

## Abstract

The differential learning approach, which includes fluctuations that occur without movement repetitions and without corrections has received growing interest in the skill acquisition field. This study aimed to determine the effects of a 9-week training intervention involving differential repeated sprint training on a series of physical tests in youth basketball players. A total of 29 participants with different maturity statuses (pre-peak height velocity (PHV), *n* = 7; mid-PHV, *n* = 6; post-PHV, *n* = 16) completed 2 sessions per week of differential repeated sprint training for a period of 9 weeks. Sessions consisted of 2 × 10 repetitions sprints of 20-m whereby participants were instructed to perform various additional fluctuations for each repetition. Before and after the training intervention, participants completed jumping tests (countermovement jump (CMJ), single-leg CMJs, the modified 505 agility test, and straight sprinting tests (0–10 splits time), and maturity status was evaluated as well. Within-group analysis showed improvement in CMJ asymmetries and changes in direction asymmetries and 10-m sprint performance for the pre-, mid-, and post-PHV groups, respectively (*p* < 0.05), with large to very large effects. Analysis of covariance demonstrated that changes in sprint time in post-PHV players were greater than in the pre- and mid-PHV groups (*p* < 0.05), with moderate effect. Adding random fluctuations during repeated sprint training appear to be a suitable and feasible training strategy for maintaining and enhancing physical performance in youth basketball players, irrespective of maturity status. Furthermore, the present findings encourage practitioners to implement the present approach in youth athletes to improve their physical performance, but they should be aware that training response can vary according to maturity status.

## 1. Introduction

The requirement for high-intensity running and longer sprint distances has increased in basketball [1]. Consequently, practitioners have been developing methods of enhancing sprint and repeated sprint ability (RSA) in team-sports athletes. Since sprinting in basketball is not exclusively straight-line, it is considered beneficial to prepare athletes to sprint in different directions and challenge the technical model. 

Nevertheless, practitioners should be aware that adolescent youth basketball players experience puberty, one period of accelerated somatic growth promoted by the synergistic effect of gonadal hormones with growth hormone and insulin-like growth factor 1 (IGF-1) [2]. During this period, several physical changes in height, weight, and strength are observed, resulting in decreased coordination and fine motor control [2]. These changes could result in increased injury risk, and individualized prevention strategies to reduce the likelihood of injury should be implemented [2,3]. This approach seems particularly imperative in “high-risk” or “load-sensitive” athletes, who simultaneously experience the period of accelerated growth and hold a high degree of sport specialization [4], such as often occurs with basketball players [5]. This is owing to the few opportunities to experience a variety of load-adaptive stimuli, resulting in fully developed neuromuscular patterns that protect against injury [4]. Nonetheless, the body of evidence suggest that underlying mechanisms to explain training adaptations are different according maturity status [6]. Whereas pre-pubertal training adaptations result primarily from nervous system development, pubertal and post-pubertal adaptations are more associated with increases in sex androgen concentrations (e.g., testosterone, growth hormone, and insulin-like growth factor) [6]. Thus, including variation during training (e.g., using differential learning principles) could be a suitable strategy for addressing individual needs, mitigating neuromuscular deficits, and reducing the chance of overloading. However, further studies are needed for better understanding of the effectiveness of this approach in youth development, whether it is related to physical performance or health related issues.

In contrast to the traditional training strategy, where fluctuations (i.e., different from biomechanical models) are viewed as errors that must be minimized, the differential learning approach [7,8] considers the fluctuations in moving systems as crucial sources for learning. A major purpose of differential learning is increasing the possibilities of movement rather than constraining them. Meanwhile, evidence has been provided that increased movement fluctuations can be quantified by the amount or structure of noise and can also be increased or modified by means of emotions [9] or fatigue [10]. Increasing noise serves to destabilize the learning system and to launch a genuine self-organizing process. In its most extreme form, differential learning includes movement variations without repetition and without correction [11]. Movement corrections in differential learning are avoided to enable the athlete to find their own optimal solution (which would not be the case if the athletes were being guided by information about “errors”). Also according to differential learning theory, increased fluctuations result in better skill acquisition and better learning rates than traditional models [12,13]. Thereby, according to the stochastic resonance principle within the differential learning theory, the noise is to be optimized rather than maximized [11].

In this regard, the benefits of the training programs based on differential learning in both technical and physical skills have been reported in team sports [14]. Related to differential sprint training, the studies by Schöllhorn et al. [12] and Arede et al. [15] are of special interest. In the first study, the effect of an intensive sprint training 5 times a week for 6 months, based on repetitions and corrections, was compared with a differential sprint training twice a week for the same duration in 2 youth male groups [12]. After 6 months, both groups improved their maximum running speed, but the differential group improved significantly more. Additionally, a pilot study of basketball-specific sprint training in differential form in female adolescents [15] provided evidence of beneficial applications in physical performance. The extent to which differential sprint training depends on the maturity level of the athletes has not yet been investigated. Based on the previous findings, including differential learning approach fluctuations in repeated sprints in a training program is assumed to have the potential for eliciting physical performance improvements. 

Therefore, the aim of this study was to examine the effect of a 9-week training intervention involving repeated differential sprint training on a series of physical tests (i.e., jumping, sprinting, and change-of-direction) but also in bilateral asymmetries, according to player maturity status. A better understanding of the effects of differential repeated sprint training on various aspects of physical performance may help practitioners to better design training tasks to improve these aspects, considering individual needs based on growth and maturation. Given the lack of previous comparable reports, we expect that repeated differential sprint training effects are independent of maturity status.

## 2. Materials and Methods

### 2.1. Participants

**Figure 1:**
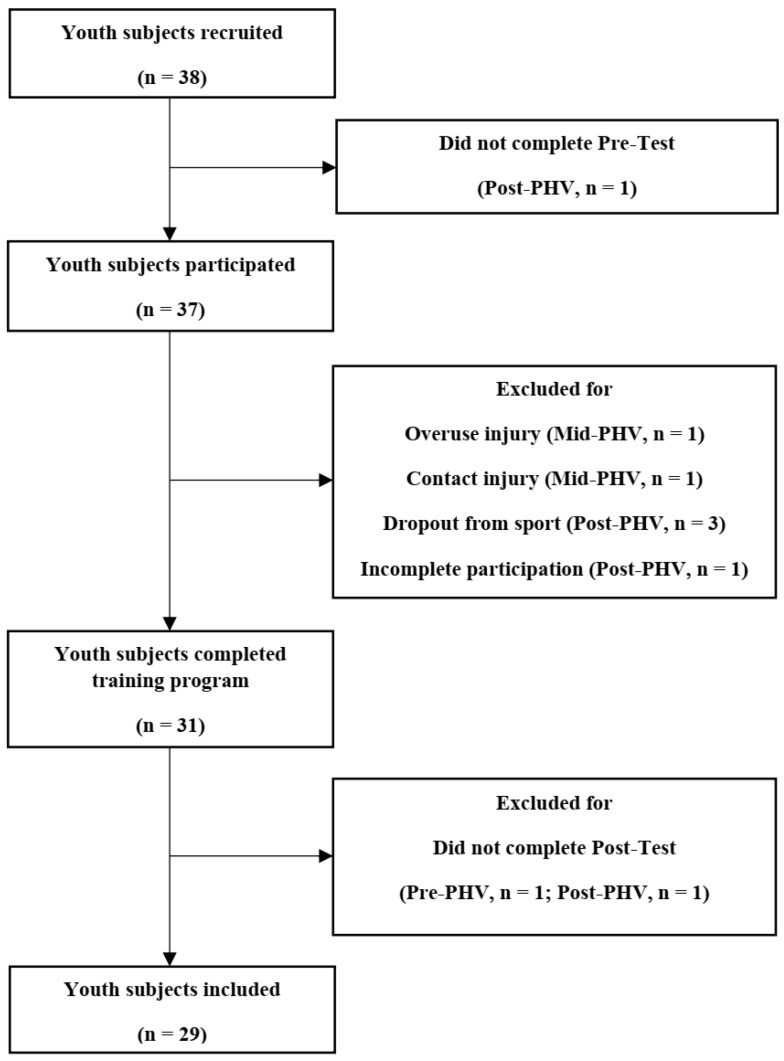
Flowchart of participant recruitment and follow-up.

A group of 38 male basketball players from the under-14 to under-18 age groups were recruited from the Portuguese Basketball Academy to participate in this study. All participants completed a total of ~270 min of basketball training (3 basketball sessions/week, 90 min/session) and 1 to 2 competitive matches per week. All participants were healthy, free of any injury within the last three months, and without previous history of injury or surgery that might have affected their physical performance. Only participants who participated in at least 90% of the workouts were considered for data analysis, which resulted in the exclusion of 1 player from post-testing analysis (Figure 1). Thirty-one players completed the training program, but only twenty-nine players were finally assessed (Figure 1). Post hoc observed power calculations (G*Power, version 3.1.9.8; University of Düsseldorf; Düsseldorf, Germany) for analysis of covariance (ANCOVA), including three groups and one covariate (α = 0.05, *d* = 0.25), revealed power (β) of 0.09. Written and informed consent was obtained from all participants’ parents, and player approval was obtained before the beginning of this investigation. The present study was approved by the Institutional Research Ethics Committee and conformed to the recommendations of the Declaration of Helsinki.

### 2.2. Procedures

This experimental study incorporated a parallel-group, repeated-measures design, whereby participants were divided into three groups with repeated sprinting training based on differential learning principles [8] (Pre-PHV, *n* = 7; Mid-PHV, *n* = 8; Post-PHV, *n* = 20). The groups were clustered according to the percentage of predicted adult height (% PAH). The training period lasted 9 weeks and was carried out within the regular in-season training sessions. The tests were performed one and two weeks before the commencement of the training period and one week after the intervention. Physical performance tests were conducted under the same experimental conditions (training session time and indoor basketball court). Testing sessions were completed on the same time interval (between 6:30 p.m. and 9:30 p.m.). A 10-min standardized warm-up was performed (5 min jogging, dynamic stretching, 10 bilateral squats, core exercises, 10 unilateral squats, and 3 vertical unilateral jumps) before all testing. Tests were conducted in the following order, respecting the principles of the *National Strength and Conditioning Association* for testing order [16]: anthropometrical measurements, jumping tests (countermovement jump (CMJ), single-leg countermovement jumps (SLCMJs), the modified 505 agility test, and straight sprinting tests (0–10 splits time). 

### 2.3. Training Program

The athletes included in the different training groups participated in two weekly training sessions during in-court training sessions (Supplementary Table S1). All the intervention drills were performed at the beginning of the training session, after the warm-up period. The differential repeated sprint training comprised 2 sets of 10 sprints for 20 m with 30 s of passive recovery between sprints and 3 min of passive recovery between sets. Before each repetition, all participants were verbally instructed by the main researcher to perform a different fluctuation (Supplementary Table S2) or a combination of fluctuations. No instructed movement fluctuation was repeated more than once in each training session. These fluctuations were selected based on previous studies involving the differential learning approach exercises for motor skills [11,15,17]. While differential learning theory, based on findings from biomechanical studies on motor learning and neuroanatomical development [18,19,20], suggests a coarse orientation on fluctuations that depend on the learning status [8], in our study, all participants, independent of their maturity status, executed the same structure of fluctuations. According to differential learning theory, beginners should focus more on varying variables that are associated with the geometry of a movement, and with advancing learning status, the focus shifts to variables that are related to velocity, acceleration, and rhythm [21]. Whether the maturity status corresponds to learning status in the investigated range of ages needs future extensive research and is beyond the scope of this study.

### 2.4. Measurements

*Somatic maturation.* Height was recorded using a commercially portable stadiometer (Tanita BF-522W, Japan, nearest 0.1 cm). Body mass was estimated using a scale (Tanita BF-522W, Japan, nearest 0.1 kg). All measurements were taken following the guidelines outlined by the International Society for the Advancement of Kinanthropometry (ISAK) by the same researcher, who holds an ISAK Level 1 accreditation. Players’ height, weight, chronological age, and mid-parent height were used to predict the adult height of each player [22]. The heights of the biological parents of each player were self-reported and adjusted for over-estimation using the previously established equations [23]. The current height of each player was then expressed as a percentage of their predicted adult height (% PAH), which can then be used as an index of somatic maturation [24]. Players were grouped into three maturity bands based on the percentage of predicted adult height attained at the time of the tournament [25]: <86% (Pre-PHV), 86–95% (Mid-PHV) and >95% (Post-PHV) of predicted adult stature (Table 1). Only for descriptive reasons, maturity timing was estimated for each player based on z-scores: average or on-time (z-score between +0.5 and −0.5), early (z-score > +0.5), and late (z-score < −0.5). 

*Bilateral and Unilateral Countermovement Jumps (CMJ)*. CMJs were assessed according to the Bosco Protocol [26]. Participants performed three successful single leg CMJs (SLCMJs) with each leg in the vertical and horizontal directions. Participants began by standing on one leg, then descended into a countermovement before extending the stance leg to jump as far or as high as possible in the vertical and horizontal directions. The landing was performed on both feet simultaneously. A successful trial included hands remaining on the hips throughout the movement and balance being maintained for at least 3 s after landing. If the trial was considered unsuccessful, a new trial was performed. In the horizontal direction, the participants started with the selected leg positioned just behind a starting line. The jump height was recorded using an infrared optical system (OptoJump Next—Microgate, Bolzano, Italy).

*The modified 505 agility test (COD).* Each participant was instructed to run to a mark situated 5 m from the starting line, perform a 180° COD using the right or left leg to push off, and return to the starting line, covering a total of 10 m [27]. The participants were asked to pass the line indicated on the ground with their entire foot at each turn. The modified 505 agility test total time was recorded with 90 cm height photoelectric cells separated by 1.5 m (Witty, Microgate, Bolzano, Italy). Each participant performed 2 sprints with COD for each side with 2 min of rest between them. Players began each trial in standing staggered position with their front feet 0.5 m behind the first timing gate. The lower limb asymmetry index (ASI) was determined using the following formula [28]: ASI = 100/Max Value (right and left)*Min Value (right and left)* − 1 + 100. The COD deficit (CODD) for the double 180° COD test for each leg was calculated via the following formula: mean double modified 505 agility test time—mean 10 m time [27].

*Sprint test.* The running speed was evaluated as 10 m (0–10 m) split time. Running times were recorded with single pairs of 90 cm high photoelectric cells separated by 1.5 m. Each participant performed 2 trials with 2 min of rest between each trial. Players began each trial in an upright standing position with their feet 0.5 m behind the first timing gate.

### 2.5. Statistical Analyses

Descriptive data are presented as mean (M) ± standard deviation (SD). The reliability of test measures was computed using an average-measures two-way random intraclass correlation coefficient (ICC) with absolute agreement, inclusive of 95% confidence intervals (CI), and the coefficient of variation (CV). The ICC was interpreted as poor (<0.5), moderate (0.5–0.74), good (0.75–0.9), or excellent (>0.9) [29]. Coefficients of variation were considered acceptable if <10% [30]. The normality of the data distribution and sphericity were confirmed using the Shapiro–Wilk statistic and Levene’s test for the equality of variances, respectively. The analysis of variance (ANOVA) with bootstrapping was used to compare the groups at baseline, and Tukey’s post hoc test was used in conjunction to examine the differences between groups. Effect sizes were evaluated using an omega squared (ω^2^), with <0.06, 0.06–0.14, and >0.14 indicating a *small, medium*, or *large* effect, respectively. A paired-samples *t*-test with bootstrapping was used to analyse within-group changes [31]. Percentage changes were calculated as ([post-training value—pretraining value]/pre-training value) × 100. Differences between pre- and post-test were calculated according to criteria described elsewhere [32]. Effect sizes (ES) of the within-group changes were evaluated using Hedges’ g correcting small sample biases [33]. The effect sizes were considered <0.2 trivial, >0.2–0.5 small, >0.5–0.8 medium, >0.8–1.3 large, and >1.3 very large [34]. An ANCOVA with Bonferroni-adjusted post hoc tests was performed to examine the differences between groups (Pre-PHV, Mid-PHV, and Post-PHV) in post-training values where the pre-training score was used as a covariate, the post-test scores as the dependent variable and the maturity status as the independent variable [35]. ES was evaluated with partial eta squared (η^2^_p_), and the threshold values were no effect (η^2^_p_ < 0.04), minimum effect (0.04 < η^2^_p_ < 0.25), moderate effect (0.25 < η^2^_p_ < 0.64), and strong effect (η^2^_p_ > 0.64) [36]. This measure has been widely cited as a measure of ES and predominantly provided by statistical software [37]. All statistical analyses were performed using the SPSS software (version 28 for Windows; SPSS Inc., Chicago, IL, USA).

## 3. Results

### 3.1. Tests Reliability

All ICCs were excellent (ICC range = 0.97–0.99), and most (5 of the 6) of the CVs were acceptable (CV range = 1.34–10.11%) (Table 2).

### 3.2. Tests Outcomes

At baseline, the training groups were significantly different in CMJ, 0–10 m sprint time, CMJR, CMJL, M505R, and M505L (*p* ≤ 0.05; large effect; see Table 3). Tukey’s post hoc analysis revealed significant differences between the Pre-PHV and Post-PHV training groups on these physical performance tests. Within-group changes for both training groups are described in Table 3. The Pre-PHV training group showed a significant decrease in CMJASY (*p* ≤ 0.05, large effect), and the Mid-PHV training group showed a significant decrease in CODASY (*p* ≤ 0.05, very large effect). Finally, the Post-PHV training group showed significant improvement in 0–10 m sprint time (*p* ≤ 0.01, large effect). According to the ANCOVA results, significant differences were observed in 0–10 m sprint time (*p* ≤ 0.05; moderate effect), with higher results for the Post-PHV than the Pre-PHV.

**Figure 2:**
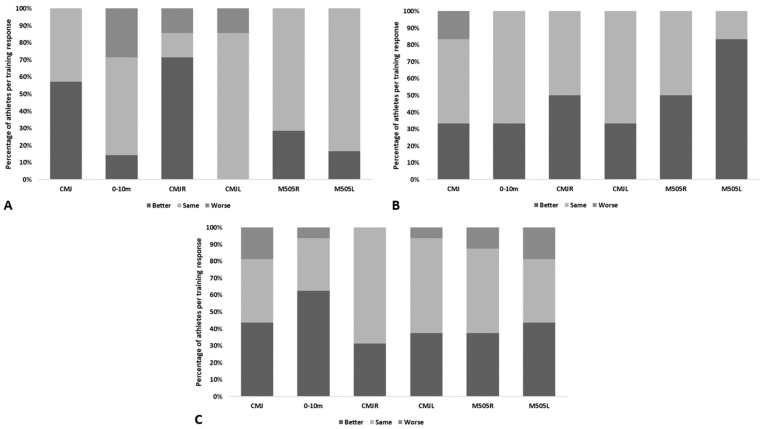
Percentage of athletes per training response. Legend: (**A**) Pre-PHV; (**B**) Mid-PHV; (**C**) Post-PHV.

Figure 2 displays the individual changes in performance from pre- to post-test for each training group. Most Pre-PHV subjects were better at CMJ (57%) and CMJR (71%) on the post-test compared with the pre-test. In the same training group, all subjects improved CMJL. Furthermore, within Pre-PHV, distinct training responses were observed for 0–10 sprint time. The majority of Mid-PHV subjects were better in M505L (50 %), when comparing to the pre-test values. However, in the same training group many subjects kept the same performance in CMJL (33%) and 0–10 sprint time (33%), in post-test. Nevertheless, in CMJ distinct training response were observed in Mid-PHV subjects. In the post-PHV group, many subjects improved 0–10 sprint time (63%); however, distinct training response was observed for different tests, with exception of CMJR.

## 4. Discussion

The aim of this study was to examine possible group specific effects of differential repeated sprinting training dependent on maturity status. Although all groups had the same training content of differential sprint training exercises, every group had a different training response in distinct variables. We found that the presented training program resulted in significant decreases in bilateral asymmetries during the physical performance tests in the Pre- and Mid-PHV subjects. Moreover, the Post-PHV training group improved their 0–10 m sprint time significantly more than the Pre-PHV subjects. Furthermore, Mid-PHV had more homogenous training responses (better and/or same), whereas more diverse training responses were observed in Pre-PHV and Post-PHV. Whether these results depend on the different levels at the beginning or on the maturity status needs further research. 

Given the lack of comparative studies on the training response by maturity status, this study should be viewed as the starting point for further studies on this topic as a further intermediate step on the way to individuality of learning [38]. The results indicate that 9 weeks of differential repeated sprinting training of adolescent male basketball players had a beneficial impact in 0–10 m sprint time in different maturity statuses, especially in Post-PHV subjects, as the effect was significantly higher than Pre-PHV. The extent to which the lower increase in performance in the Pre-PHV group is indicative of either too much variation or wrong variations for the performance level and thus suggests that a more traditional approach or individually adapted variations to sprint training are recommended, which at this level still has sufficient variation for optimal learning even with repetition. Whether there is a principle level dependency, needs to be clarified in future studies [11,39]. However, performance advances should not forget the long-term development of athletes, where other parameters like higher symmetry in CMJs could be a preventive and precondition for further performance gains. The beneficial impact in 0–10 m sprint time is in line with previously results obtained in a pilot study on female basketball players [15]. Nonetheless, results from other studies differ in magnitude. For example, 6 weeks of plyometric training resulted less effective improvement in 0–10 m sprint time in youth basketball players [40], whereas other short- to medium-term training protocols (combined strength and conditioning, small-sided games training, high-intensity interval training, and plyometric, strength and change-of-direction training) were more effective at improving the 0–10 m sprint time in pubertal youth basketball players, based in their maturity offset [41,42,43]. In contrast, the current protocol gives indication to be superior to 6-week eccentric overload training that was direction-specific [44], and 10-week strength training program with random recovery times [45] to achieve gains in 0–10 m sprint time. These findings are in line with Rumpf and colleagues [46] who suggested that other methods (e.g., plyometrics and strength) can be more effective to improve speed during puberty, whereas the combination of methods in athletes with accumulated training and well-developed training skills combination of training methods, forms and purposes in a single drill can be particularly effective [47]. Thereby, discrepancies between studies, and between maturity status can have substantial influence on neuromotor development aspects which underlie possible training adaptations to the differential repeated sprint training. However, with respect to the actual more generally discussed replication crisis [48,49] and the critical discussion of the applied statistics therein the results are rather to be considered as helpful proposals and cannot be generalized [50]. Including our own investigation, they at best provide suggestions that it is worthwhile to conduct further research in this area.

During differential repeated sprinting training, many alternating variants of sprinting occur in a single session. In this regard, in comparison with normal sprinting patterns, differential sprints provide a multitude of kinematic and kinetic changes [12,51,52,53,54,55,56]. Here, the general idea of differential learning theory is to also use restrictions in one area to increase fluctuations in another area in the short term and then increase the number of opportunities in general in the long term by combining the again released constrained area with the increased fluctuations in the other areas. Thereby it is important to notice that the restrictions are explicitly not used for guiding the system towards an externally given problem solution but to initiate a self-organizing process. For example, sprinting with the arms held across the chest or running with the arms held behind the back resulted in increased peak lateral ground reaction forces and higher peak hip internal rotation, and knee flexion [54]. Moreover, forward trunk lean sprinting resulted in greater lengths of all the three hamstring muscles at foot strike and toe-off [53]. Evidence was also provided that the restriction of scapula movement influenced the stance-leg motion and whole-body position during the first step, but also the sprint speed [55]. Restricted arm action (i.e., crossed arms) resulted in compensatory upper body motions that could provide the rotational forces needed to offset the lower body angular momentum generated by the swinging legs [52]. Adding “erroneous” and non-representative -movements by increasing the existing fluctuation during repeated sprint training generate short term co-contractions (i.e., simultaneous contraction of agonist and antagonist muscles around a joint), which provide more joint stability and higher accelerating forces [57,58]. However, combining higher levels of noise, speed, and co-contractions may reduce the momentary speed of movement but provide stronger and movement adequate stimuli for muscle groups that are requested in situations of high competitive stress [56,59]. Greater physical performance requires a balance between maximizing the movement intensity, controlling movement through co-contractions, faster relaxation, and reducing muscle slack [56,59]. In this regard, the transient shift from protective, long-latency reflexes to pre-active, short-latency reflex recruitment throughout maturation, particularly the reduction of inhibitory mechanisms to protect the Musculo-tendon unit [60] can result in more efficient stretch shortening cycle (SSC) actions [58], explaining better training response in 0–10 m sprint time of Post-PHV subjects comparing to the Pre-PHV. 

Closely connected to the increase in the multitude of muscle activation patterns due to changed joint lever conditions and, consequently, due to the changed proprioception are changes in the brain activation. Neurophysiological adaptations resulting from differential learning include electroencephalographic frequencies in the alpha- and theta-bands which benefits short-term memory and learning [61]. Moreover, there is evidence that differential learning results in increased theta activity in contralateral parieto–occipital regions [61] but also stimulates the somatosensory and motor system and engages more regions of the cortex [62]. Notwithstanding these meaningful findings, brain activity after differential learning has been only analyzed in young adults, and the effects of differential learning, including the brain activity, may be different during neurodevelopment in childhood and puberty, resulting in inter-individual differences in terms of physical performance. Indeed, during young adulthood occurs a peaking of white matter volume [63], an area which controls the signals that neurons share, coordinating how well brain regions work together [64]; whereas, the peak of grey matter (i.e., area with large number of neurons) volume occurs before typical age of puberty onset [63]. Moreover, the process of myelination (i.e., acquisition of the highly specialized myelin membrane around axons) occurs from the back of the cerebral cortex to front, and from subcortical regions to higher centers of the central nervous system (e.g., cerebellum and cortex) [65]. This suggests that the learning of complex skills may lead to distinct neurological adaptations with respect to the maturity status. In fact, learning complex skills results in noticeable changes in the white matter; however, learning after adolescence is associated with increased white matter development in regions that are still undergoing myelination, such as the forebrain [64]. This region integrates different brain areas, such as the prefrontal cortex, the premotor cortex, and the primary motor cortex associated with voluntary movement [65]. Altogether, Post-PHV may have benefited from both brain maturity patterns and neurophysiological adaptations of training in specific brain areas, resulting in improved performance during voluntary actions, such as short sprinting.

After the differential repeated sprint training program, irrespective of maturity status, participants displayed higher values of unilateral vertical jumping (except for CMJ_L_ in pre-PHV) compared to the pre-test values. Similar benefits for unilateral vertical jumping were observed in a pilot study [15]; whereas different effects occurred after a group of youth basketball players completed different training programs [44,45,66]. Albeit enhanced neuromuscular qualities can be achieved using movement variability [67], overload and assist musculature of hip and knee regions involved in the SSC may be beneficial (e.g., higher peak activity of knee stabilizers muscles or considering concentric peak vertical power/body weight) [68,69], to have higher unilateral jumping height in youth athletes. 

Furthermore, differential repeated sprint training program was particularly beneficial for Mid-PHV athletes regarding vertical unilateral jumping. These results are particularly promising because using the present training strategy, practitioners can simultaneously achieve positive adaptations resulting from natural improvements in maximal muscular power during puberty [60], but also adjust load patterns considering particularities of accelerated growth period. Thereby, during puberty muscle strength increases, but there is no increase in proportion to limb inertial properties; and, excessive physical loading may cause skeletal injury, particularly through overuse mechanism. Therefore, jumping training during puberty should be carefully prescribed because the increased risk of joint overload, and coordination training (movement adaptability) should be particularly emphasized [2]. In this regard, practitioners can provide a relatively safe, enjoyable, and effective training program more based on individual needs, frequently alternating many variants of sprinting in a single session using differential learning, resulting in improved multifaceted adaptability, and consequently improved vertical unilateral jumping. Moreover, subjects may benefit from an immediate transfer to specific sport such as previously observed after differential learning based jumping training in handball [21]. 

In addition, it is widely established that change-of-direction speed is an essential skill among youth athletes engaging in team sports [70]. The current training program was beneficial (but without statistical significance) during the agility test including 180° change of direction. This change of direction involves a more aggressive cutting angle (≥75°) which includes higher braking requirements [71]. In this regard, fastest performance in 180° change of direction includes higher propulsive and braking forces (particularly horizontal) on the final foot contact [72], and has been associated with higher eccentric and isometric strength [70]. Moreover, the 180° change of direction involves high peak muscle activity of the knee stabilizers (vastus medialis and lateralis) which play a key role in frontal play control [69]. Thus, chronic exposure to frequently alternating variants of sprinting in a single session can generate structural and functional adaptations which positively influences biomechanical determinants of 180° change of direction, resulting in improved performance in a controlled setting. Furthermore, youth athletes may have benefited from continued neural development and hormonal changes throughout childhood and adolescence, resulting in improved change of direction performance [73]. Nevertheless, a previous study involving 16 years old male basketball players which included multidirectional eccentric overload training resulted in similar gains in the same 180° change of direction test [44]. Thus, older players may benefit from multidimensional adaptations (i.e., biomechanical, morphological, and neuromuscular levels) resulting from eccentric training [74], which could provide an advantage in high-intensity actions, such as cutting. Moreover, athletes may have benefited from performing resistance exercises (i.e., unilateral lateral eccentric overload training) including frontal and transverse plane-dominated tasks, similar to a 180° COD test. Notwithstanding, the present findings are promising because the training strategy is low in cost due to no equipment requirements and the results are beneficial in 180° change of direction, including the potential of increased neurophysiological adaptations [61]. 

Increases in bilateral asymmetries are observed during early stages of adolescence or in the period of accelerated growth, particularly when rapid gains in limb length occur [75]. In this regard, young athletes can be more predisposed to various injuries in high-intensity activities (e.g., cutting and landings), because of additional stress placed on the weaker leg due to bilateral asymmetry [75]. In the present study, most of subjects had CMJ_ASY_ above 10% cut-off criterion for bilateral asymmetries becoming more likely to have an injury. Indeed, the participants of our study had larger CMJ_ASY_ and COD_ASY_ than previously observed in youth tennis players, irrespective of maturity status based in maturity offset [76]. Notwithstanding, the applied training strategy was effective to decrease CMJ_ASY_ and COD_ASY_, in Pre- and Mid-PHV, respectively. In this regard, differential repeated sprint training which generates neurophysiological adaptations seems to be similarly beneficial to other methods to reduce discrepancies between lower limbs (e.g., bilateral and unilateral strength and plyometric training, and balance and core training) [77], in young subjects where neural mechanisms are mainly responsible for training adaptations [47]. On the contrary to previously observed after 10-week strength training program with random recovery times involving post-pubertal male basketball players [45], the decrease in CMJ_ASY_ was substantially lower in the present study. This comparison between studies suggests that resistance training may be more effective to induce positive changes in CMJ_ASY_. 

In addition, contrary to what was observed in the pilot study, probably one reason of a lower starting performance level [15], the present protocol did not result in increased CMJ performance. In previous studies including young male basketball players, CMJ values showed higher improvements after completing different short- to medium-term training programs (most of them including jumps) compared to that of the present study, irrespective of maturity status [41,42,43,78,79,80,81]. It appears that a greater dynamic correspondence of CMJ with different exercises (vertical jumps, axial based resistance exercises, etc.) may be responsible for achieving the CMJ improvement. Also, in older basketball players (≥16 years old), short- to medium resistance training programs (6–10 week) in unilateral and bilateral fashion resulted more [45,82], albeit lower magnitude was observed in direction-specific eccentric overload training [44]. It suggests different between-studies underlying mechanisms explaining the adaptations in jumping performance, particularly in pre- and pubertal stages, where higher improvements were observed. Thereby, the adaptative response to frequently alternating variants of sprinting in a single session using differential learning which result in improved ability to use the positive effect of SSC to the vertical jumping performance, could be related to neural improvements in these stages [47]. Notwithstanding, in prepubertal and pubertal stages, the magnitude of improvement in a key physiological mechanism underlying efficient movement, such as utilization of the SSC, seems to be greater according the level of neuromuscular load experienced in this plane, how occurs in plyometric training [60].

## 5. Conclusions

Often interpreted at a first superficial glance as arbitrary variations, on a slightly closer look the variations proposed under the differential learning approach turn out to be targeted interventions for a holistic neuromuscular and specific training that must be adjusted to every discipline and level of performance [8]. Through constantly changing postures within the context of the discipline (crossed arms in front of the body, arms above the head, etc.) there is not only a stronger tuning of targeted muscle groups through correspondingly changed levers, but also a more versatile or noisy tuning of the neuronal system (e.g., motor and somatosensory cortices), which thus becomes more robust against future disturbances. Thereby, the body and especially head rotations around various axis are of special importance since they train the versatile interactions of the vestibular apparatus with the activating and perceiving apparatus [20,56]. Our findings indicate that adding “erroneous” fluctuation and non-representative movements during repeated sprint training can result in a significant reduction of bilateral asymmetries during physical performance tests, in pre- and mid-PHV basketball players. Furthermore, Post-PHV athletes improved their 10-m sprint to a greater extent than Pre- and Mid-PHV. Generally, the Mid-PHV had more positive and homogenous training response (better and/or same), whereas more varied response was observed in their Pre- and Post-PHV counterparts. Indeed, the inclusion of these fluctuations within repeated sprint training may positively influence the basketball players’ movement patterns towards more effective and stabilized skills. The positive adaptations are potentially owing to concomitant neurophysiological adaptations induced by the differential repeated sprint training. Nonetheless, how much of the learning progress is influenced by the continuously changing biomechanical conditions and how much by the cognitive effect of not having errors corrected in connection with the accompanied disadvantageous brain activations needs to be clarified in future. Nevertheless, the present findings may encourage practitioners to implement similar protocols in youth athletes to improve physical performance, although always being aware that training response can be variable according to maturity status. Furthermore, the higher variability of stimuli during the training also suggests looking for additional effects on prevention of injuries, which have higher incidence during periods of accelerated growth. Finally, further studies should examine the real differences between the application of differential repeated sprint training to the natural development without any training protocol (i.e., control group), advancing towards more individuality in training.

## Figures and Tables

**Table 1:** Descriptive data of the subjects (Mean ± SD).

Variables	Pre-PHV (*n* = 7)	Mid-PHV (*n* = 6)	Post-PHV (*n* = 16)
Biological age (years)	12.01 ± 0.36	13.32 ± 0.58	16.97 ± 1.15
Height (cm)	149.14 ± 7.31	160.67 ± 7.99	179.31 ± 8.68
Body mass (kg)	39.86 ± 10.78	53.83 ± 10.80	74.13 ± 15.09
PAH (%)	83.71 ± 1.11	88.67 ± 2.50	98.69 ± 1.70
Timing	−0.01 ± 0.50	1.75 ± 0.63	0.90 ± 0.39
Maturity Timing (Z-score)	Early = 1 On-time = 4 Late = 2	Early = 6 On-time = 0 Late = 0	Early = 14 On-time = 2 Late = 0
Training experience (years)	4.86 ± 0.38	3.50 ± 1.38	5.94 ± 2.79

Legend: PHV = Peak of height velocity; PAH = Percentage of Adult Height. Note: A z-score < −0.5 is late, > +0.5 is early, and between +0.5 and −0.5 is average or on-time.

**Table 2:** Reliability data for test variables. Data are presented as value with lower- and -upper confidence limits.

Test Variables	ICC (95% CL)	CV (%) (95% CL)
CMJ (cm)	0.98 (0.97; 0.99)	5.66 (3.81; 7.52)
0–10 m (s)	0.99 (0.98; 0.99)	1.34 (0.88; 1.80)
CMJ_R_ (cm)	0.98 (0.97; 0.99)	8.19 (6.47; 9.92)
CMJ_L_ (cm)	0.98 (0.97; 0.99)	10.11 (7.82; 12.39)
M505_R_ (s)	0.97 (0.92; 0.98)	2.58 (2.01; 3.14)
M505_L_ (s)	0.98 (0.95; 0.99)	1.96 (1.36; 2.56)

Abbreviations: ICC = Intraclass correlation coefficient; CV = Coefficient of variation; CL = Confidence limits; CMJ = Countermovement jump height; 0–10 m = 0–10 m sprint time; M505 = Modified 505 agility test; R = Right; L = Left.

**Table 3:** Inferences of the training programs intervention on subject’s performance measures.

Variables		Pretest, Mean ± SD	Postest, Mean ± SD	∆ %	*p*	Hedge’s g	Between-Groups Pretest Differences (*p*)	ω^2^	ANCOVA (*p*)	η^2^_p_
CMJ (cm)	Pre-PHV	22.06 ± 6.55	23.44 ± 5.41	6.28	0.171		**0.007** *	0.26 (−0.04; 0.47)	0.466	
	Mid-PHV	25.23 ± 6.80	26.37 ± 6.63	4.49	0.444					
	Post-PHV	34.73 ± 9.95	35.51 ± 8.61	2.23	0.372					
0–10 m (s)	Pre-PHV	2.18 ± 0.29	2.17 ± 0.28	−0.46	0.731		**0.014** *	0.22 (−0.06; 0.43)	**0.020** *	0.27
	Mid-PHV	2.07 ± 0.16	2.03 ± 0.17	−2.25	0.138					
	Post-PHV	1.88 ± 0.21	1.81 ± 0.17	−3.53	**0.000**	1.05 (0.42; 1.65)				
CMJ_R_ (cm)	Pre-PHV	11.74 ± 3.90	12.44 ± 3.99	5.96	0.222		**0.014** *	0.22 (−0.06; 0.44)	0.072	
	Mid-PHV	12.28 ± 2.79	14.22 ± 3.21	15.74	0.205					
	Post-PHV	19.66 ± 8.01	20.86 ± 6.35	6.14	0.052					
CMJ_L_ (cm)	Pre-PHV	13.67 ± 3.61	13.61 ± 4.17	−0.42	0.945		**0.027** *	0.18 (−0.07; 0.40)	0.332	
	Mid-PHV	12.72 ± 2.27	14.82 ± 2.93	16.51	0.131					
	Post-PHV	19.67 ± 7.45	20.85 ± 7.64	6.01	0.098					
CMJ_ASY_ (%)	Pre-PHV	26.96 ± 6.61	19.67 ± 7.56	−27.04	**0.046**	0.88 (−0.03; 1.75)	0.195		0.095	
	Mid-PHV	27.57 ± 12.77	26.57 ± 8.28	−3.62	0.819					
	Post-PHV	20.33 ± 9.96	18.61 ± 11.34	−8.47	0.611					
M505_R_ (s)	Pre-PHV	3.15 ± 0.35	3.06 ± 0.38	−2.90	0.112		**0.026** *	0.18 (−0.07; 0.40)	0.430	
	Mid-PHV	3.07 ± 0.17	3.01 ± 0.18	−1.90	0.321					
	Post-PHV	2.78 ± 0.32	2.72 ± 0.22	−2.22	0.164					
M505_L_ (s)	Pre-PHV	3.13 ± 0.36	3.10 ± 0.38	−0.91	0.365		**0.023**	0.19 (−0.00; 0.41)	0.177	
	Mid-PHV	3.15 ± 0.13	3.00 ± 0.22	−4.56	0.063					
	Post-PHV	2.80 ± 0.33	2.73 ± 0.25	−2.41	0.091					
COD_ASY_ (%)	Pre-PHV	4.85 ± 4.05	5.54 ± 2.19	14.42	0.648		0.594		0.326	
	Mid-PHV	6.34 ± 1.62	4.02 ± 1.41	−36.56	**0.015**	1.94 (0.50; 3.32)				
	Post-PHV	5.33 ± 2.18	5.68 ± 2.96	6.43	0.705					
CODD_R_ (s)	Pre-PHV	1.03 ± 0.14	0.94 ± 0.13	−8.97	0.172		0.192		0.664	
	Mid-PHV	1.04 ± 0.17	0.99 ± 0.12	−4.94	0.326					
	Post-PHV	0.93 ± 0.14	0.94 ± 0.10	1.00	0.804					
CODD_L_ (s)	Pre-PHV	0.99 ± 0.13	0.96 ± 0.10	−2.88	0.541		0.115		0.864	
	Mid-PHV	1.09 ± 0.12	0.97 ± 0.14	−10.70	0.075					
	Post-PHV	0.95 ± 0.14	0.93 ± 0.11	−1.19	0.782					

Abbreviations: CMJ = Countermovement jump height; 0–10 m = 0–10 m sprint time; M505 = Modified 505 agility test; COD = Change of direction test; CODD = COD deficit; R = Right; L = Left; ASI = Bilateral asymmetry; * Pre-PHV vs. Post-PHV (*p* < 0.05).

## Data Availability

The data that support the findings of this study are available from the corresponding author, J.A., upon reasonable request.

## Supplementary Materials

The following supporting information can be downloaded at: https://www.mdpi.com/article/10.3390/ijerph191912265/s1, Table S1: Training program variations. Table S2: Examples of the fluctuations performed during differential repeated sprint training interventions.
