# Supplementary material for: Differential Repeated Sprinting Training in Youth Basketball Players: An Analysis of Effects According to Maturity Status
Source: Int J Environ Res Public Health. 2022 Sep 27;19(19):12265. doi: 10.3390/ijerph191912265 (PMC9566386; doi:10.3390/ijerph191912265)
Supplement: Supplementary file 1 [file ijerph-19-12265-s001.zip › Table S1.pdf]

Table S1. Training program variations.

**Week**		1	2	3	4	5	6	7	8	9
**Starting type**		Regular	Regular	Regular	Regular	Rolling	Rolling	Rolling	Rolling	Regular
**Finishing type**		Regular	Regular	Regular	Jump	Regular	Jump	COD	Turn	Dec
**Variations**	**Day 1**	37	66	34	43	41	29	5	28	73
		78	64	36	3	8	11	17	46	49
		10	69	18	28	54	19	68	86	78
		46	55	6	21	72	86	67	64	27
		50	70	22	2	71	32	51	55	63
		28	82	19	15	84	80	41	32	11
		71	23	79	70	15	78	24	82	68
		25	23	35	57	55	4	5	48	55
		51	85	34	56	71	68	57	47	22
		27	49	54	67	82	64	79	37	81
		72	3	61	36	67	33	77	15	33
		9	43	7	80	42	58	70	33	14
		60	16	71	10	43	51	56	12	74
		77	40	1	66	67	28	3	1	72
		15	34	51	75	61	78	24	83	1
		44	80	64	31	41	60	81	43	25
		11	18	8	41	68	12	71	84	86
		17	21	45	55	30	14	48	71	13
		65	61	42	79	33	10	14	65	70
		80	85	12	63	44	18	69	60	59
	**Day 2**	66	25	64	16	54	34	21	39	43
		64	81	43	82	26	21	5	76	69
		71	23	6	69	63	38	18	70	5
		21	19	63	76	45	6	78	72	34
		48	1	37	61	38	53	57	83	22
		57	5	5	17	2	77	52	51	73
		76	40	39	13	74	66	82	58	16
		62	70	27	11	16	52	7	30	36
		74	48	62	74	41	57	68	65	65
		24	55	38	57	75	58	12	86	86
		59	2	82	14	48	56	25	52	76
		63	15	16	51	24	67	29	28	70
		54	83	75	77	84	42	21	1	32
		20	80	19	25	4	7	6	27	72
		3	72	30	24	55	54	27	60	24
		4	8	43	83	34	3	77	29	10
		62	18	61	79	81	72	64	25	71
		22	27	23	30	72	40	47	36	45

		77	61	2	6	35	66	54	56	38
		60	17	55	2	22	68	30	52	60

Note: The full description of each number is available at table 2. Abbrevations: Dec = Deceleration.
