# Supplementary material for: Differential Repeated Sprinting Training in Youth Basketball Players: An Analysis of Effects According to Maturity Status
Source: Int J Environ Res Public Health. 2022 Sep 27;19(19):12265. doi: 10.3390/ijerph191912265 (PMC9566386; doi:10.3390/ijerph191912265)
Supplement: Supplementary file 1 [file ijerph-19-12265-s001.zip › Table S2.pdf]

Table S2. Examples of the fluctuations performed during differential repeated sprint training interventions.

#	Body part	Fluctuations	#	Body part	Fluctuations
1	Head	Head back	53	Scapula	Scapula elevated
2		Head forward	54		Right scapula elevated
3		Head back and forward	55		Left scapula elevated
4		Head rotated to the left	56		Scapula retracted
5		Head rotated to the right	57		Right scapula retracted
6		Head rotated left and right	58		Left scapula retracted
7		Head tilted to the left	59		Scapula depressed
8		Head tilted to the right	60		Right scapula depressed
9		Head tilted to the right and left	61		Left scapula depressed
10		Head circumduction to the left	62		Scapula protracted
11		Head circumduction to the right	63		Right scapula protracted
12	Eyes	Right eye closed	64		Left scapula protracted
13		Left eye closed	65	Trunk	Trunk rotation to the left
14		Blinking right eye	66		Trunk rotation to the right
15		Blinking left eye	67		Trunk rotation to the right and left
16		Blinking eyes	68		Trunk tilted laterally to the left
17		Look to the right	69		Trunk tilted laterally to the right
18		Look to the left	70		Trunk tilted laterally to the left and right
19		Look to the right + Blinking eyes	71		Trunk tilted back
20		Look to the left + Blinking eyes	72		Trunk tilted forward
21		Cover left eye with left hand	73		Trunk tilted back and forward
22		Cover left eye with right hand	74	Hands	Hands on hip
23		Cover right eye with left hand	75		Right hand on hip
24		Cover right eye with right hand	76		Left hands on hip
25		Look to the right + Cover left eye with left hand	77		Hands behind head
26		Look to the right + Cover left eye with right hand	78		Hands on forehead
27		Look to the right + Cover right eye with left hand	79		Right hand behind head
28		Look to the right + Cover right eye with right hand	80		Left hand behind head
29		Look to the left + Cover left eye with left hand	81		Hands behind back
30		Look to the left + Cover left eye with right hand	82		Right hand behind back
31		Look to the left + Cover right eye with left hand	83		Left hand behind back
32		Look to the left + Cover right eye with right hand	84		Clapping ahead
33		Simultaneous swing straigth arms	85		Clap behind back
34		Alternated swing straigth arms	86		Clap front and back
35	Arms	Static two arms up			
36		Two arms up with hand pronation and supination			
37		Two arms close to the torso			
38		Simultaneous forward arm rotation			
39		Simultaneous backward arm rotation			
40		Alternated forward arm rotation			
41		Alternated backward arm rotation			
42		Simultaneous arm abduction and adduction			
43		Alternated arm abduction and adduction			
44		Arms open to the side			
45		Arms open down			
46		Crossed arms			
47		Arms stretched forward			
48		Arms stretched back			
49		Right arm up + left arm down			
50		Left arm up + right arm down			
51		Left arm up + right arm to the side			
52		Right arm up + left arm to the side			
